# Expression of Soluble Form of Aurora A as a Predictive Factor for Neoadjuvant Therapy in Breast Cancer Patients: A Single-Center Pilot Study

**DOI:** 10.3390/cancers15225446

**Published:** 2023-11-16

**Authors:** Pawel Winter, Malgorzata Fuksiewicz, Agnieszka Jagiello-Gruszfeld, Zbigniew Nowecki, Beata Kotowicz

**Affiliations:** 1 Breast Cancer and Reconstructive Surgery Department, Maria Sklodowska-Curie National Research Institute of Oncology, 02-781 Warsaw, Poland; pawel.winter@nio.gov.pl (P.W.); agnieszka.jagiello-gruszfeld@nio.gov.pl (A.J.-G.); zbigniew.nowecki@nio.gov.pl (Z.N.); 2 Cancer Biomarker and Cytokines Laboratory Unit, Maria Sklodowska-Curie National Research Institute of Oncology, 02-781 Warsaw, Poland; malgorzata.fuksiewicz@nio.gov.pl

**Keywords:** breast cancer, neoadjuvant treatment, predictive marker, AURKA, Aurora A, HER3, ErbB3, thymidine kinase 1, TK1

## Abstract

**Simple Summary:**

AURKA tissue expression has been shown to be a predictive and prognostic factor in many solid tumors, including breast cancer. To improve the outcome of breast cancer (BC) treatment, further personalization and the exploration of the use of new biomarkers is needed to assess the effects of the applied treatment. New predictive markers may reduce the cost and toxicity of therapy. In our single-center prospective study, we determined the serum levels of Aurora A, thymidine kinase 1, and human epidermal growth factor receptor type 3 (HER3) using ELISA kits in 119 women with BC before neoadjuvant treatment (NAT). We showed that in a biologically heterogeneous group of BC patients, the pretreatment serum Aurora A levels were of significant value in predicting the response to NAT. The response to NAT was assessed in the postoperative material after systemic treatment as the pCR, that is, an indicator of the complete pathological response to neoadjuvant treatment. To our knowledge, this is the first study of the predictive value of serum AURKA in patients with breast cancer.

**Abstract:**

Purpose: To search for new predictive breast cancer biomarkers. We analyzed the serum concentrations of biomarkers involved in carcinogenesis, which can also be targeted by therapy. Methods: In a single-center prospective study, the serum levels of Aurora A, thymidine kinase 1, and human epidermal growth factor receptor type 3 (HER3) were determined in 119 women with BC before neoadjuvant treatment using ELISA kits. Results: The following clinical data were analyzed: age; TNM; the expression of ER, PGR, HER2, and Ki67; histological grade (G); and the response to neoadjuvant treatment (NAT) in the residual tumor burden classification (RCB). A complete pathological response (pCR) was achieved after NAT in 41 patients (34%). The highest proportion of the patients with a confirmed pCR was found for triple negative breast cancer (TNBC) (62.5%); non-luminal HER2-positive (52.6%) cancer subtypes (*p* = 0.0003); and in the G3 group (50%; *p* = 0.0078). The patients with higher levels of Aurora A were more likely to achieve pCR (*p* = 0.039). In the multivariate analysis, the serum Aurora A levels ≥ 4.75 ng/mL correlated with a higher rate of pCR (OR: 3.5; 95% CI: 1.2–10.1; *p* = 0.023). Conclusions: We showed that in a biologically heterogeneous group of BC patients, the pretreatment serum Aurora A levels were of significant value in predicting the response to NAT.

## 1. Introduction

Breast cancer (BC) is the most common malignant neoplasm and the leading cause of cancer death among women worldwide. It is estimated that, in 2020, over 2.26 million new cases of BC among women were diagnosed worldwide, and almost 685,000 women died from this cancer [1]. BC is a heterogeneous disease with a very complex biology. Based on the expression of the tissue markers estrogen receptor (ER), progesterone receptor (PR), epidermal growth factor receptor type 2 (HER2), and mitotic index—Ki67, invasive BC is classified into five main biological subtypes: luminal A (LA), luminal B (LB), luminal B HER2-enriched (HER2-LB), non-luminal HER2-positive (HER2-NL), and triple negative breast cancer (TNBC). Tissue markers play both prognostic and predictive roles in the management of BC.

Neoadjuvant treatment (NAT) should be considered in all patients who may be eligible for systemic treatment. There are no proven differences in the overall survival (OS) and disease-free survival (DFS) between NAT and adjuvant treatment [2]. NAT is an important tool for reducing staging and treatment response monitoring. The pathological complete response (pCR) archived after NAT has a proven beneficial long-term effect on the DFS and OS, especially in TNBC- and HER2-positive cancers [2]. There are different pathologic pCR reporting systems—the AJCC-TNM, B18, Miller–Payne, modified Nottingham scale, Pinder, and Residual Cancer Burden (RCB), which is preferred in clinical research applications [3]. RCB is an independent prognostic factor for DFS and OS; moreover, it can select patients with a high risk of recurrence and poor prognosis [4,5,6]. Despite the significant advances in recent years, treatment results are still not satisfactory, and it is unknown why some patients with the same cancer subtype benefit from treatment and others do not. One of the newly available and validated prognostic tools are multi-parameter genetic tests, e.g., Oncotype DX and Mammaprint, which can be used as independent predictors in the qualification to adjuvant treatment [7,8]. These genetic tests require formalin-fixed paraffin-embedded tumor tissue samples; moreover, genetic testing is expensive and not widely available. The ideal biomarker should have a high sensitivity and specificity, could be determined at any stage of treatment in a cheap, easily accessible, non-invasive way, and the obtained results should be reproducible and objective. Many of these criteria may be met by potential peripheral blood (PB) markers. PB can be easily divided into fractions containing selected groups of substances. After the centrifugation of whole blood with the addition of a clotting activator, serum is obtained, which may contain potential markers—proteins, metabolites, lipids, autoantibodies, miRNA, and ctDNA [9]. In the past, researchers have selected several serum BC markers such as CA 15-3, CEA, tissue antigen polypeptide (TPA), and tissue specific polypeptide antigen (TPS). Unfortunately, due to low sensitivity and specificity, none of them are currently recommended in BC management [10,11,12,13]. Therefore, new breast cancer biomarkers are still being explored. One concept is the analysis of enzymes involved in the regulation of the cell cycle, carcinogenesis, angiogenesis, and cell adhesion and migration, which could also be a target for anti-cancer therapies. Based on a review of the literature in recent years, it seems that the study of the biomarkers AURKA A, HER3, and TK1, which are native to tumor cells and exhibit prooncogenic activity, may be in keeping with the current clinical needs. Most of the available studies are concerned with the tissue expression of biomarkers; whereas, in our study, we evaluated the serum concentrations of the soluble forms of these proteins. Recently, new therapeutic strategies, including neoadjuvant treatment, have been introduced into practice; however, there is a lack of tools to evaluate the effects of treatment as well as the adverse effects of NAT. The proposal that measuring the concentrations of these biomarkers will be helpful in identifying a group of patients who will not benefit from the use of NAT, which is extremely important, also in terms of the toxicity of this treatment (neuro-, cardio- and nephrotoxicity), in the future, may influence therapeutic decisions. More personalization is needed to improve the outcome of breast cancer (BC). New predictive markers may reduce the cost and toxicity of therapy.

Aurora A (AURKA) belongs to the AURORA serine-threonine kinases. In mammalian cells, three different AURORA kinases were discovered—A, B, and C, the main function of which is, in general, the regulation of the cytoskeleton, chromosomes, and cell division processes. The findings on the role of AURKB in cancer development have led to the development of small molecule AURKB inhibitors as a potential therapeutic strategy for cancer treatment [14]. Elevated Aurora-B expression is often associated with several types of cancer, including breast cancer. However, it is unclear whether this alteration contributes to the tumor response to therapies and prognosis [15]. The role of AURKC in cancer is not fully understood and requires further research. AURKA has the most significant role in oncology. AURKA is a protein that regulates the cell cycle process. Regardless of the kinase function, AURKA in the cell nuclei shows the activity of a transcription factor and participates in the assembly and stabilization of the replisome [16]. AURKA enhances the phenotype of neoplastic stem cells, increases their survival, and their ability to migrate and invade [17]. AURKA expression shows tissue specificity (testes, skeletal muscles, thymus, spleen); increased levels are found in the colon, breast, ovarian, cervical, neuroblastoma, prostate, and bladder cancer cells. The expression of AURKA expression can be considered as a neoplastic marker in breast cancer [18]. Due to its functions, AURKA has been the subject of many oncology research studies. Its overexpression in breast cancer cells has been shown to be associated with resistance to hormone therapy with kinase inhibitors and worsening prognosis and overall survival [19]. Other work has found that AURKA expression in tumor tissue in obese and TNBC patients is associated with a worse prognosis and higher recurrence rates [20]. Currently, AURKA inhibitors are being investigated and the utility of anti-AURKA therapy in cancer treatment is being explored [21]. AURKA is analyzed for its usefulness as a tumor marker in breast cancer. A study compared tissue proliferative markers in breast cancer and demonstrated the superior prognostic value of AURKA compared to Ki67 [22]. Few articles attempt to evaluate the clinical value of determining AURKA concentrations in the serum of breast cancer patients.

Human epidermal growth factor receptor 3 (HER3) is a transmembrane tyrosine kinase receptor (EGFR), and is a 180 kDa glycoprotein encoded by the ERBB3 gene at locus 12q13 [23]. In addition to HER3, three other proteins have been identified: HER1, HER2, and HER4. HER3 consists of an extracellular domain, a transmembrane portion, and an intracellular domain [24]. In addition to the 180 kDa glycoprotein, the alternative splicing of the ERBB3 gene also produces p45 and p85 glycoproteins, which lack a transmembrane portion and intracellular domain, are easily secreted outside the cell and are determined as soluble ErbB3 (sErbB3) [25]. The naturally occurring secreted form of the human ErbB3 receptor, p85-soluble ErbB3 (sErbB3), is a strong negative regulator of the heregulin (HRG)-stimulated activation of ErbB2, ErbB3, and ErbB4. p85-sErbB3 binds to HRG with an affinity comparable to that of full-length ErbB3 and competitively inhibits high-affinity HRG binding to ErbB2/ErbB3 heterodimers on the surface of breast cancer cells. p85-sErbB3 inhibits the HRG-induced phosphorylation of ErbB2, ErbB3, and ErbB4 in breast cancer-derived cell lines [26]. To demonstrate intracellular activity and signal transduction, HER3 requires heterodimer formation with other proteins of the EGFR group, e.g., HER2. HER2/HER3 heterodimers show oncogenic activity by activating intracellular pathways such as (PI-3K)/AKT, MAPK, RAS-ERK, which stimulate proliferation, angiogenesis, migration, and increase the survival of neoplastic cells [27,28]. In some patients with resistance to anti-HER2 therapy, elevated levels of neuregulins are observed. According to studies, the pathways that activate HER3 to form HER2/HER3 heterodimers may play a key role in the mechanism of resistance of HER2-positive tumors to trastuzumab treatment and the resistance of LB tumors to hormone therapy [29]. HER3 has been shown to be overexpressed in breast, bladder, ovarian, prostate, melanoma, and neuroblastomas [30]. HER3 has been strongly overexpressed in the metastatic foci of breast cancer, especially in the brain. HER3 overexpression is also associated with resistance to hormone therapy, anti-HER2 therapy, and taxanes. HER3 inhibitors are being investigated in cancer treatment [31]. In recent years, methods for determining HER3 in serum have been described for further research [32]. Secreted ErbB3 has been developed as a serum biomarker for determination using human monoclonal antibodies in ELISA assays.

Thymidine kinase 1 (TK1) is a cell cycle phase-dependent enzyme involved in DNA synthesis, encoded by the TK1 gene at locus 17q25.3. Thymidine kinase exists in cells as two isoenzymes: TK1 and TK2. TK1 is involved in the thymidine phosphorylation reaction, which is a necessary step in its incorporation into DNA. TK2 is found in the mitochondria. In cancer cells, TK1 activity may remain elevated throughout the cell cycle. TK1 is released into the extracellular space from cells that die during division, which is characteristic of neoplastic cells [33]. Plasma TK1 activity has been found to be higher in patients with hematologic cancers, breast, lung, colon, stomach, ovarian, and cervical cancer, and may serve as a proliferative marker [34]. In solid tumors, the serum levels of TK1 have been shown to correlate with the stage of the disease. High levels of TK1 in breast cancer have been shown to correlate with a poorer OS and PFS [35]. He Q et al. found that, in breast cancer, increased serum TK1 levels determined 3 months after surgery indicated a higher rate of recurrence [36]. Thymidine kinase 1 in serum predicts an increased risk of distant or locoregional recurrence after surgery in patients with early breast cancer [36]. Another study evaluated the plasma TK1 activity in patients with diffused luminal breast cancer on palbociclib therapy after 2 weeks of therapy and proved that a decrease in TK1 activity correlates with a better prognosis and response to treatment [37]. A study demonstrated the usefulness of monitoring serum TK1 assays in correlation with tumor size as an indicator of the number of damaged cells during neoadjuvant treatment; an increase in concentrations after treatment initiation was correlated with PCR after therapy achievement [38]. The use of TK1 as a target for cancer therapy is also being analyzed.

The purpose of this study is to assess whether the determination of selected biomarkers prior to treatment can be useful to predict the response to neoadjuvant therapy used in patients with breast cancer.

## 2. Materials and Methods

### 2.1. Ethical Statement

The research protocol was approved by the Bioethics Committee at the Maria Sklodowska-Curie National Research Institute of Oncology in Warsaw, Poland; on the date of 29 July 2019 (authorization No. 34/2019). We performed a single-center prospective observational study according to the ethical standards of the Declaration of Helsinki. The samples were taken after informed consent was obtained from all study participants.

### 2.2. Patients

The initial study population was a randomized group of 252 women diagnosed with breast cancer and scheduled for treatment at the Breast Cancer Unit (BCU), Department of Breast Cancer and Reconstructive Surgery. Baseline clinical data (before treatment) were collected and analyzed: age, ER, PR, Ki67, and HER2 expression, biological subtype, histological grade of malignancy according to NGS classification, UICC/AJCC 8. Clinical classification of TNM.

Inclusion criteria for our study:

1. Age above 18 years.
2. Patients with a confirmed diagnosis of breast cancer on histopathological examination from a core needle biopsy.
3. Qualified for NAT treatment by MDT (multidisciplinary team) within BCU according to ESMO and PTO (Polish oncological society) recommendations for neoadjuvant treatment.
4. With complete medical records and results to assess the response to NAT in the RCB classification.
5. Given informed consent to participate in the study.

Exclusion criteria:

1. Lack of MDT qualification for neoadjuvant treatment.
2. Lack of documentation, which prevents the assessment of response to treatment.

The response to NAT was evaluated in the postoperative material after systemic treatment as pCR, that is, an indicator of complete pathological response to neoadjuvant treatment. The absence of tumor cells in the removed tissue section indicated a complete response to NAT. Only patients who received NAT and had complete medical records, which allowed for the evaluation of response to neoadjuvant treatment, were qualified for further study. The NAT scheme is used depending on the biological subtype and clinical indications. A group of 119 patients was qualified for the next part of the study and marker determination. The control group consisted of 47 randomly selected healthy women. Sera were obtained and secured up to 14 days before the start of treatment.

### 2.3. Methods

Blood was collected from patients prior to treatment and then centrifuged at 2600 rpm at 15 min. Separated serum samples were frozen immediately after collection and stored at 70 °C in aliquots until the time of analysis. To detect ErbB, AURKA, and ThK1 levels in serum samples, we used the commonly used method of protein detection, the enzyme-linked immunosorbent assay (ELISA). In a sandwich ELISA, the plate is coated with the human recombinant monoclonal antibody against the desired antigen (AURKA, HER3, ThK), the sample is added and then the detection antibody is allowed to bind to any captured antigen. After incubation and washing away of excess reagents, a polyclonal antibody conjugated to the marking enzyme was added. After reincubation and removal of excess reactants, the substrate solution of hydrogen peroxide and chromogen tetramethylbenzidine was added in the next step. The enzymatic reaction was stopped by adding 2N sulfuric acid. The concentrations of the biomarkers tested were determined by measuring optical density, on a spectrophotometer, at a wavelength of 450 nm. Parameters tested were determined twice using the enzyme-linked immunosorbent assay (ELISA) method: Human ERBB3 ELISA KIT by Biorbyt Ltd., Cambridge, UK; Human hThK 1—by EIAAB SCIENCE Inc., Wuhan, China; Human AURKA (Aurora A) Wuhan Fine Biotech Co., Ltd., Wuhan, China.

#### Statistical Analysis

SPSS Statistics version 23 by IBM was used for statistical calculations. Continuous variables of biomarker concentrations were categorized. The cutoffs were chosen to divide the patients into three equal groups. In the first step, a one-way analysis was performed using the χ^2^ test of independence, and in the second step, a multivariate analysis using a logit regression model with the pCR as the dependent variable. The model was built based on the following classical clinical variables that can affect response to treatment: TNM stage, malignancy grade, and the status of receptors defining the biological subtype: ER, PR, HER2, and Ki67. The fitted model was used to test the predictive value of the investigated markers. In the modeling process, the method of stepwise elimination of variables with standard levels of inclusion (*p* < 0.05) and exclusion (*p* > 0.1) was used. Additionally, the distributions of the biomarkers analyzed in women with breast cancer and healthy women were compared and their diagnostic potential was evaluated. ROC curves were used to assess the diagnostic sensitivity and specificity of the markers, pairwise non-parametric comparisons of the neighboring areas under curves (AUC) were performed by the Wilcoxon test.

Marker concentrations and age of the patients were analyzed using the Spearman rank correlation test. The adopted level of statistical significance α = 0.001. Next, χ^2^ tests were carried out to characterize the concentrations of the markers tested in relation to clinical data. The concentration cut-offs were chosen to divide patients into three equal groups, which offers the best chance of detecting relationships between test variables. Taking into account the Bonferroni correction for multiple tests, individual tests were performed at the level of statistical significance equal to 0.001.

## 3. Results

### 3.1. Patient Characteristics

**Figure 1:**
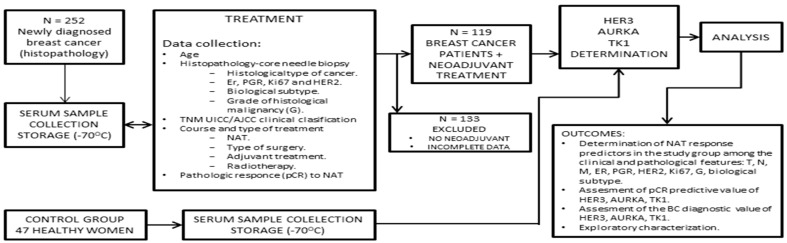
Study design.

Overall, 119 women were qualified for the determination of marker concentrations (Figure 1).

The median age and the interquartile range were 53 (42–63) years. Tumors up to 5 cm in size (T1 and T2) were diagnosed in 81 patients, and no metastases were detected in the regional lymph nodes (N0) in 52 patients. At the time of the beginning of treatment, eight women had stage IV (M1) disease, of which one with TNBC achieved pCR. Additionally, a patient progressed during NAT. In total, eight patients did not receive surgical treatment and were classified in the non-pCR group. In 111 women, the disease was diagnosed with an intermediate and high histological grade of NGS (G2 and G3). The largest group of patients were the patients with luminal neoplasms (37 LB and 13 LA), and, in 45 cases, the expression of the HER2 receptor was detected. The patients received neoadjuvant treatment according to individual clinical indications. In the study group, 44 patients (HER2-LB; HER2-NL) received chemotherapy according to the regimen of TCH/TCH + P (docetaxel 75 mg/m^2^, carboplatin AUC5-6, trastuzumab 8 mg/kg +/− pertuzumab 840 mg) as a neoadjuvant therapy (36.9%), 61 (51.26%) received sequential treatment (TNBC; LB; LA)−4xAC + 12xPXL (4x(doxorubicin 60 mg/m^2^ + cyclophosphamide 600 mg/m^2^) + 12x paclitaxel 60 mg/m^2^), and 14 patients who were LA or LB received hormonal treatment (11.76%). Forty-one (34.45%) patients achieved pCR, who were further analyzed with clinical characteristics by the χ^2^ test (*p* < 0.05). The highest percentage of patients with confirmed pCR was found in the G3 group (50%; *p* = 0.0078). The relationship was shown for the biological subtype, with the highest percentage of patients with pCR found for the subtypes positive for TNBC (62.5%) and non-luminal HER2 subtypes (52.6%) subtypes (*p* = 0.0003). There was no correlation between the pCR rates and tumor size in the TNM classification (*p* = 0.3584), or the status of the lymph nodes (*p* = 0.2435). The detailed characteristics of the study group and the results of the pCR are presented in Table 1.

The concentrations of the investigated markers were determined in the blood samples of the patients collected before the initiation of NAT and the control group. The ROC curves were designated to estimate the diagnostic value of HER3, AURKA, and TK1. The areas under the ROC curves were, respectively, for HER3—AUC = 0.634 (95% CI: 0.524–0.745), for AURKA—AUC = 0.453 (95% CI: 0.351–0.555) and for TK1—AUC = 0.847 (95% CI: 0.771–0.923). The fields for HER3 and TK1 differed statistically significantly from 0.5; *p* values for HER3—*p* = 0.020; for TK1—*p* < 0.001. 

### 3.2. Univariate Analysis

**Figure 2:**
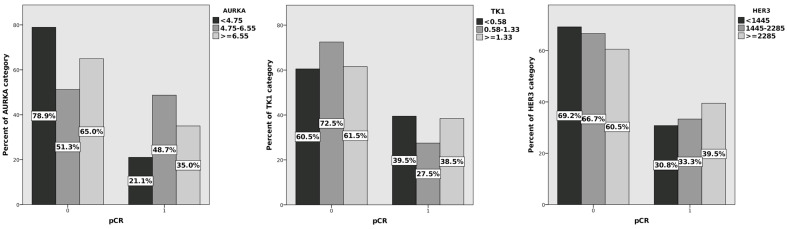
Percentage of pCR and biomarker concentrations in selected cut-off levels, univariate analysis: AURKA, TK1, HER3.

The univariate analysis using the χ^2^ independence test was performed to correlate the pCR with the marker serum levels. The concentration cut-offs were chosen to divide patients into three equal groups, which provides the best chance of detecting a relationship between the pCR and the test variables. A statistically significant relationship was found between the pCR and the AURKA concentration (*p* = 0.039). No similar relationship was found for HER3 (*p* = 0.712) and TK1 (*p* = 0.466). The complete pathological response rates for the AURKA levels (<4.75 ng/mL; 4.75–6.66 ng/mL; ≥6.55 ng/mL) were, respectively, 21.1%, 48.7%, and 35.0%. The results of the univariate analysis are presented in Figure 2.

### 3.3. Multivariate Analysis

**Figure 3:**
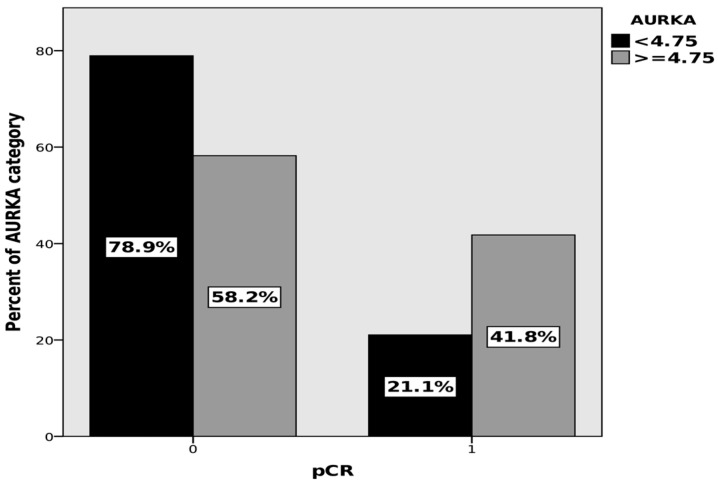
Pathological complete response (pCR) rates depending on AURKA concentrations, multivariate analysis *p* = 0.023, OR: 3.5 (95% CI: 1.2–10.1).

In the next step, a multivariate analysis was performed using a logit regression model with pCR as the dependent variable. The model was built based on clinical parameters that may affect response to treatment, namely, the progression level of TNM, the histological grade G, and the status of receptors defining the biological subtype of the tumor, ER, PR, HER2, and Ki67. In the multivariate analysis, the effect of the AURKA concentration ≥ 4.75 ng/mL on the probability of achieving pCR was found (*p* = 0.023), OR: 3.5 (95% CI: 1.2–10.1). The second and third categories of the AURKA variable were combined because of the lack of a statistically significant difference between them. The complete pathological response rates for values less than and greater than or equal to 4.75 ng/mL are presented in Figure 3.

A statistically significant effect on the pCR was also found for the status of an N0 lymph node status (*p* = 0.039), no PR expression (*p* < 0.001), and Ki67 > 20% (*p* < 0.025). The respective odds ratios (with 95% confidence interval) were for N0—0.503 (95% CI: 0.263–0.965), for PR (-)—0.104 (95% CI: 0.038–0.284) and for Ki67 > 20%—5.44 (95% CI: 1.24–23.9). Table 2 presents the parameters of the adopted logit model.

### 3.4. Exploratory Analysis

The non-parametric correlation showed a statistically significant positive relationship between the concentration of AURKA and TK1 *p* < 0.001; the Spearman’s correlation coefficient was 0.318, indicating a weak correlation. With the level of statistical significance adopted, no other correlations were found.

There was no significant correlation between the biomarker concentrations and clinical characteristics: the tumor size, lymph node metastases, presence of distant metastases, expression of tumor tissue markers—ER, PR, HER2, and Ki67, biological subtype, grade of histological malignancy.

## 4. Discussion

Breast cancer, despite significant improvements in treatment outcomes, remains the leading cause of cancer death in women worldwide [1]. In recent years, a number of significant studies have emerged on the development of screening tools or prognostic factors using a variety of blood biomarkers such as proteins, autoantibodies, miRNAs, metabolites, and lipids in breast cancer patients. The use of mass spectroscopy has identified a panel of five proteins that, with high sensitivity and specificity, can differentiate the serum of women with breast cancer from healthy individuals and predict recurrence-free survival in women with ER-negative tumors [39]. Other researchers have attempted to summarize recent findings on the use of a variety of biomarkers assayed using different methods as new tools for BC detection, as well as discuss the limitations that must be overcome before using specific markers [9]. However, biomarkers for BC screening are still in the early stages of development, and various clinical and preclinical issues need to be resolved before these biomarkers can be used clinically [40]. In addition to screening, it is also an important problem to find biomarkers that, when determined prior to treatment, would be a significant prognostic factor for patients with BC, which was the aim of our study. By analyzing the progress of knowledge on breast cancer to date, it is easy to conclude that the milestones in improving treatment are discoveries in the pathophysiology of tumor cells, allowing for the differentiation of tumor biology and the implementation of personalized therapies [41,42].

Many intensive studies are being carried out to investigate new potential entry points for anticancer therapies [43,44]. It is very important to convert laboratory observations into clinical applications. There is a need to further develop our knowledge of tumor cell metabolism, division, and migration processes and the mechanisms leading to resistance to current treatments. In this process, the further differentiation of breast cancer phenotypes using predictive biomarkers is essential. It is necessary to discover markers that allow the rapid and noninvasive characterization of the disease, also in the metastasis phase, due to the different biology of metastatic foci [45,46]. Markers determined in peripheral blood can provide many of these goals.

The response to NAT was evaluated according to the RCB classification, which was a replacement end point for DFS and OS, due to the short observation period (a median of 29 months), which was confirmed by the literature [47]. In our study, there is a relatively high percentage of pCR obtained in all patients, irrespective of the stage and therapy used. The highest percentage of pCR was obtained in patients with TNBC and HER2-NL tumors. Similar results have previously been described in patients who received neoadjuvant treatment according to the regimens used in our clinic [48].

In our study, we found that as histological grade G increases, the percentage of pCR increases. Furthermore, the highest percentages of pCR were found in patients with triple negative tumors and with HER2 receptor expression. On the other hand, in the multivariate analysis, a statistically significant result was obtained in obtaining pCR with the N0 trait, the lack of PR expression, and Ki67 > 20%. Livingston-Rosanoff et al. analyzed data from 38,864 women from the National Cancer Database who received neoadjuvant chemotherapy for breast cancer between 2010 and 2013. The complete pathological response was defined as ypT0, and the status of the lymph nodes was not considered. The dependence of the pCR obtained after NAT on the tumor size and biological subtype was demonstrated; significantly higher percentages were obtained in the T1–T2 patients and in the TNBC and HER2-NL groups, similar to our results [49]. Our results were consistent with the study by Katayama et al., who observed the highest percentage of pCR in NAT in the group of 500 patients with the HER2-NL subtype and with histological grade G3 [50].

Analyzing the concentrations of the selected biomarkers in this study’s group of BC patients, the highest diagnostic sensitivity was found for CT 1. Thymidine kinase is an essential enzyme in the process of DNA synthesis and repair. TK1 is released from cells that break down when they divide, a phenomenon typical of cancer. After being released into the extracellular space, TK1 polymerizes to form complex protein complexes of different mass and activity, depending on the tissue from which it comes [51]. However, the given methods show different sensitivity depending on the masses of the tested TK1 complexes, which influences the results obtained. It has also been shown that, in the case of solid tumors—breast and prostate cancer—a large percentage of TK1 is released into the serum in an inactive form [51]. An additional problem in assessing the activity of TK1 is the presence of TK1 inhibitors in serum [52]. Due to the limitations mentioned in the techniques to measure TK1 activity, attempts were made to develop methods based on the detection of TK1 with specific antibodies [53]. Studies have shown an adverse effect of high concentrations of TK1 on the OS and DFS [35,54,55]. Our study showed that there is no correlation between the pCR after NAT and serum TK1 concentration. We found a significantly higher TK1 in the control group than in BC; it should be considered that, in our cohort, TK1 is not a diagnostic marker in breast cancer.

Based on data from the literature presenting the relationship between HER3 overexpression and the progression and development of chemoresistance in patients with malignant neoplasms, we evaluated the usefulness in clinical practice of determining the concentration of HER3 in the blood serum of patients with breast cancer. In our test group, high diagnostic sensitivity was observed for HER3 concentrations. However, we did not demonstrate any correlation of serum HER3 concentrations with the pathological response after neoadjuvant treatment. Due to alternative splicing, HER3 occurs in the human body in the form of three isoforms: p180, p85, and p45, which can be detected in peripheral blood by ELISA [32,56]. Commercially available ELISA kits differ from each other in the type of HER3-specific antibodies used, which can affect the results obtained [32]. There are studies that show the different role of individual HER3 isoforms in the pathology of neoplasms. Li et al. demonstrated a key role for the intracellular domain of HER3 in the pathways that promote the colonization and proliferation of TNBC metastases within the bone [57]. The importance of individual HER3 isoforms in breast cancer biology has not been studied so far. Although HER3 plays an extremely important role in cancer pathophysiology and resistance mechanisms to current anti-HER2 therapy, due to the lack of standardization of the test methods, the presence of isoforms with different biological significance and low tissue specificity, the use of serum HER3 is currently limited [24,58].

To our knowledge, this is the first study of the predictive value of serum AURKA in patients with breast cancer. In the study group of patients, we demonstrated a relationship between the AURKA concentrations and NAT response. AURKA concentrations greater than 4.75 ng/mL were associated with a higher chance of achieving pCR.

The importance of Aurora A kinase tissue overexpression in the pathophysiology of cancer is the subject of many studies, and AURKA is a promising target for anticancer therapies [59]. AURKA tissue expression has been demonstrated to be a predictive and prognostic factor in many solid tumors. AURKA overexpression in BC cells is associated with resistance to hormone therapy, taxanes, kinase inhibitors, and the deterioration of prognosis and overall survival [18,19,60]. Other studies have found that AURKA expression in TNBC and obese patients is associated with a worse prognosis and a higher relapse rate [20]. Standards for assessing tissue AURKA overexpression have not yet been developed, and one of the key limitations of the research is the different methodology, making it impossible to objectively compare the results.

It is commonly known that the current standard tissue marker for BC is Ki67, which is an independent predictor and prognostic factor. High tissue expression of Ki67 is likely to predict a higher response rate to neoadjuvant treatment, regardless of the type of treatment: hormone therapy, chemotherapy, or targeted therapy. It is recommended to search for a similar universal serum proliferative marker for breast cancer that could distinguish between the groups of patients who would benefit from NAT. Being able to estimate the benefit of NAT before it is initiated is very important in terms of both the toxicity of the therapy and in economic terms. In our pilot study, we showed that AURKA could be a potential predictive marker for NAT. Since we did not show an association of its concentrations with the biological subtype of BC and the type of therapy, it seems that AURKA A may be an independent and universal parameter relevant to estimate the benefit of NAT in BC patients. The study requires confirmation in a larger group of patients. In the future, it is also planned to evaluate the OS and PFS of patients participating in this study 5 and 10 years after treatment, to determine the prognostic value of serum biomarkers.

## 5. Conclusions

In a single-center pilot study, we showed that in a biologically heterogeneous group of breast cancer patients, the pretreatment serum Aurora A levels may be of significant value in predicting the response to NAT.

## Figures and Tables

**Table 1:** Patient characteristics and pCR outcomes.

Characteristics	N = 119 (%)	pCR	
		% of Patients	*p*
Age (median, range)	53 (31–77)		
ER positive	74 (62.18%)		
PR positive	61 (51.26%)		
HER2 positive	45 (37.82%)		
Ki67 ≤ 20	23 (19.33%)		
Ki67 > 20	96 (80.67%)		
Treatment			
TCH/TCH + P *	44 (36.97%)		
4xAC + 12xPXL **	61 (51.26%)		
Hormonal	14 (11.76%)		
Tumor size (T)			
T1	8 (6.72%)	5 (62.50%)	0.3584
T2	73 (61.34%)	25 (34.25%)	
T3	25 (21.01%)	7 (28.00%)	
T4	11 (9.24%)	4 (36.25%)	
Tx	2 (1.68%)	0 (0%)	
Lymph node status (N)			
N0	52 (43.70%)	21 (40.38%)	0.2435
N1	44 (36.97%)	15 (34.09%)	
N2	19 (15.97%)	3 (15.78%)	
N3	4 (3.36%)	2 (50.00%)	
Distance metastasis status (M)			
M0	111 (93.28%)	40 (34.48%)	0.1670
M1	8 (6.62%)	1 (12.50%)	
Tumor grade (G)			
G1	8 (6.72%)	1 (12.50%)	0.0078
G2	61 (51.26%)	15 (24.59%)	
G3	50 (42.02%)	25 (50.00%)	
Biological subtype			
Luminal A	13 (10.92%)	0 (0%)	0.0003
Luminal B	37 (31.09%)	6 (16.66%)	
Luminal B HER2-enriched	26 (21.85%)	10 (38.46%)	
Non-Luminal HER2-positive	19 (15.97%)	10 (52.63%)	
TNBC /triple negative	24 (20.17%)	15 (62.50%)	

* TCH/TCH + P (docetaxel 75 mg/m^2^, carboplatin AUC5–6, trastuzumab 8 mg/kg +/− pertuzumab 840 mg), ** 4xAC + 12xPXL − 4x(doxorubicin 60 mg/m^2^ + cyclophosphamide 600 mg/m^2^) + 12xpaclitaxel 60 mg/m^2^.

**Table 2:** Parameters of the adopted logit model.

Parameters	β Coefficient	Standard Error	*p* Value	Odds Ratio	95%—Confidence Interval	
					Lower	Upper
N0 *	−0.687	0.332	**0.039**	**0.503**	0.263	0.965
PR (-) **	−2.259	0.511	**<0.001**	**0.104**	0.038	0.284
Ki67 > 20%	1.694	0.755	**0.025**	**5.441**	1.239	23.893
AURKA ≥ 4.75 ng/mL	1.244	0.545	**0.023**	**3.470**	1.192	10.105
Constant	−3.178	1.546	0.040	0.042		

* N0—no node metastasis, ** PR (-)—no PR expression.

## Data Availability

Data are contained within the article.

## References

[1] Sung H., Ferlay J., Siegel R.L., Laversanne M., Soerjomataram I., Jemal A., Bray F. (2021). Global cancer statistics 2020: GLOBOCAN estimates of incidence and mortality worldwide for 36 cancers in 185 countries. CA Cancer J. Clin..

[2] Early Breast Cancer Trialists’ Collaborative Group (EBCTCG) (2018). Long-term outcomes for neoadjuvant versus adjuvant chemotherapy in early breast cancer: Meta-analysis of individual patient data from ten randomised trials. Lancet Oncol..

[3] Provenzano E., Bossuyt V., Viale G., Cameron D., Badve S., Denkert C., MacGrogan G., Penault-Llorca F., Boughey J., Curigliano G. (2015). Standardization of pathologic evaluation and reporting of postneoadjuvant specimens in clinical trials of breast cancer: Recommendations from an international working group. Mod. Pathol..

[4] Müller H.D., Posch F., Suppan C., Bargfrieder U., Gumpoldsberger M., Hammer R., Hauser H., Dandachi N., Prein K., Stoeger H. (2019). Validation of Residual Cancer Burden as Prognostic Factor for Breast Cancer Patients After Neoadjuvant Therapy. Ann. Surg. Oncol..

[5] Symmans W.F., Wei C., Gould R., Yu X., Zhang Y., Liu M., Walls A., Bousamra A., Ramineni M., Sinn B. (2017). Long-Term Prognostic Risk After Neoadjuvant Chemotherapy Associated with Residual Cancer Burden and Breast Cancer Subtype. J. Clin. Oncol..

[6] Campbell J.I., Yau C., Krass P., Moore D., Carey L.A., Au A., Chhieng D., Giri D., Livasy C., Mies C. (2017). Comparison of residual cancer burden, American Joint Committee on Cancer staging and pathologic complete response in breast cancer after neoadjuvant chemotherapy: Results from the I-SPY 1 TRIAL (CALGB 150007/150012; ACRIN 6657). Breast Cancer Res. Treat..

[7] Duffy M.J., O’Donovan N., McDermott E., Crown J. (2016). Validated biomarkers: The key to precision treatment in patients with breast cancer. Breast.

[8] Naito Y., Urasaki T. (2018). Precision medicine in breast cancer. Chin. Clin. Oncol..

[9] Loke S.Y., Lee A.S.G. (2018). The future of blood-based biomarkers for the early detection of breast cancer. Eur. J. Cancer.

[10] Seale K.N., Tkaczuk K.H.R. (2022). Circulating Biomarkers in Breast Cancer. Clin. Breast Cancer.

[11] Jassem J., Krzakowski M. (2018). Breast cancer. Oncol. Clin. Pract..

[12] Lin D.C., Genzen J.R. (2018). Concordance analysis of paired cancer antigen (CA) 15-3 and 27.29 testing. Breast Cancer Res. Treat..

[13] Van Poznak C., Somerfield M.R., Bast R.C., Cristofanilli M., Goetz M.P., Gonzalez-Angulo A.M., Hicks D.G., Hill E.G., Liu M.C., Lucas W. (2015). Use of Biomarkers to Guide Decisions on Systemic Therapy for Women with Metastatic Breast Cancer: American Society of Clinical Oncology Clinical Practice Guideline. J. Clin. Oncol..

[14] Borah N.A., Reddy M.M. (2021). Aurora Kinase B Inhibition: A Potential Therapeutic Strategy for Cancer. Molecules.

[15] Zhang Y., Jiang C., Li H., Lv F., Li X., Qian X., Fu L., Xu B., Guo X. (2015). Elevated Aurora B expression contributes to chemoresistance and poor prognosis in breast cancer. Int. J. Clin. Exp. Pathol..

[16] Guarino Almeida E., Renaudin X., Venkitaraman A.R. (2020). A kinase-independent function for AURORA-A in replisome assembly during DNA replication initiation. Nucleic Acids Res..

[17] Zheng F., Yue C., Li G., He B., Cheng W., Wang X., Yan M., Long Z., Qiu W., Yuan Z. (2016). Nuclear AURKA acquires kinase-independent transactivating function to enhance breast cancer stem cell phenotype. Nat. Commun..

[18] Lykkesfeldt A.E., Iversen B.R., Jensen M.B., Ejlertsen B., Giobbie-Hurder A., Reiter B.E., Kirkegaard T., Rasmussen B.B. (2018). Aurora kinase A as a possible marker for endocrine resistance in early estrogen receptor positive breast cancer. Acta Oncol..

[19] Wander S.A., Cohen O., Gong X., Johnson G.N., Buendia-Buendia J.E., Lloyd M.R., Kim D., Luo F., Mao P., Helvie K. (2020). The Genomic Landscape of Intrinsic and Acquired Resistance to Cyclin-Dependent Kinase 4/6 Inhibitors in Patients with Hormone Receptor–Positive Metastatic Breast Cancer. Cancer Discov..

[20] Jiang J., Guo Z., Xu J., Sun T., Zheng X. (2020). Identification of Aurora Kinase A as a Biomarker for Prognosis in Obesity Patients with Early Breast Cancer. Onco Targets Ther..

[21] Lin X., Xiang X., Hao L., Wang T., Lai Y., Abudoureyimu M., Zhou H., Feng B., Chu X., Wang R. (2020). The role of Aurora-A in human cancers and future therapeutics. Am. J. Cancer Res..

[22] Ali H.R., Dawson S.J., Blows F.M., Provenzano E., Pharoah P.D., Caldas C. (2012). Aurora kinase A outperforms Ki67 as a prognostic marker in ER-positive breast cancer. Br. J. Cancer.

[23] Yarden Y., Sliwkowski M.X. (2001). Untangling the ErbB signalling network. Nat. Rev. Mol. Cell Biol..

[24] Mishra R., Alanazi S., Yuan L., Solomon T., Thaker T.M., Jura N., Garrett J.T. (2018). Activating HER3 mutations in breast cancer. Oncotarget.

[25] Memon A.A., Gilliver S.C., Borre M., Sundquist J., Sundquist K., Nexo E., Sorensen B.S. (2018). Soluble HER3 predicts survival in bladder cancer patients. Oncol. Lett..

[26] Lee H., Akita R.W., Sliwkowski M.X., Maihle N.J. (2001). A naturally occurring secreted human ErbB3 receptor isoform inhibits heregulin-stimulated activation of ErbB2, ErbB3, and ErbB4. Cancer Res..

[27] Miricescu D., Totan A., Stanescu-Spinu I.-I., Badoiu S.C., Stefani C., Greabu M. (2020). PI3K/AKT/mTOR Signaling Pathway in Breast Cancer: From Molecular Landscape to Clinical Aspects. Int. J. Mol. Sci..

[28] Black L.E., Longo J.F., Carroll S.L. (2019). Mechanisms of Receptor Tyrosine-Protein Kinase ErbB-3 (ERBB3) Action in Human Neoplasia. Am. J. Pathol..

[29] Menendez J.A., Mehmi I., Papadimitropoulou A., Vander Steen T., Cuyàs E., Verdura S., Espinoza I., Vellon L., Atlas E., Lupu R. (2020). Fatty Acid Synthase Is a Key Enabler for Endocrine Resistance in Heregulin-Overexpressing Luminal B-Like Breast Cancer. Int. J. Mol. Sci..

[30] Mizuno T., Kojima Y., Yonemori K., Yoshida H., Sugiura Y., Ohtake Y., Okuma H.S., Nishikawa T., Tanioka M., Sudo K. (2020). Neoadjuvant chemotherapy promotes the expression of HER3 in patients with ovarian cancer. Oncol. Lett..

[31] Karachaliou N., Lazzari C., Verlicchi A., Sosa A.E., Rosell R. (2017). HER3 as a Therapeutic Target in Cancer. BioDrugs.

[32] D’Abronzo L.S., Pan C.X., Ghosh P.M. (2018). Evaluation of protein levels of the receptor tyrosine kinase ERBB3 in serum. Methods Mol. Biol..

[33] Jagarlamudi K.K., Shaw M. (2018). Thymidine kinase 1 as a tumor biomarker: Technical advances offer new potential to an old biomarker. Biomark. Med..

[34] Weagel E.G., Burrup W., Kovtun R., Velazquez E.J., Felsted A.M., Townsend M.H., Ence Z.E., Suh E., Piccolo S.R., Weber K.S. (2018). Membrane expression of thymidine kinase 1 and potential clinical relevance in lung, breast, and colorectal malignancies. Cancer Cell Int..

[35] Wang Z., Zhang W., Huo B., Dong L., Zhang J. (2020). Relationship between thymidine kinase 1 before radiotherapy and prognosis in breast cancer patients with diabetes. Biosci. Rep..

[36] He Q., Fornander T., Johansson H., Johansson U., Hu G.Z., Rutqvist L.E., Skog S. (2006). Thymidine kinase 1 in serum predicts increased risk of distant or loco-regional recurrence following surgery in patients with early breast cancer. Anticancer Res..

[37] Bagegni N., Thomas S., Liu N., Luo J., Hoog J., Northfelt D.W., Goetz M.P., Forero A., Bergqvist M., Karen J. (2017). Serum thymidine kinase 1 activity as a pharmacodynamic marker of cyclin-dependent kinase 4/6 inhibition in patients with early-stage breast cancer receiving neoadjuvant palbociclib. Breast Cancer Res..

[38] Tribukait B. (2020). Early prediction of pathologic response to neoadjuvant treatment of breast cancer: Use of a cell-loss metric based on serum thymidine kinase 1 and tumour volume. BMC Cancer.

[39] Chung L., Moore K., Phillips L., Boyle F.M., Marsh D.J., Baxter R.C. (2014). Novel serum protein biomarker panel revealed by mass spectrometry and its prognostic value in breast cancer. Breast Cancer Res..

[40] Kazarian A., Blyuss O., Metodieva G., Gentry-Maharaj A., Ryan A., Kiseleva E.M., Prytomanova O.M., Jacobs I.J., Widschwendter M., Menon U. (2017). Testing breast cancer serum biomarkers for early detection and prognosis in pre-diagnosis samples. Br. J. Cancer.

[41] Zeillinger R., Kury F., Czerwenka K., Kubista E., Sliutz G., Knogler W., Huber J., Zielinski C., Reiner G., Jakesz R. (1989). HER-2 amplification, steroid receptors and epidermal growth factor receptor in primary breast cancer. Oncogene.

[42] Doroshow D.B., Doroshow J.H. (2020). Genomics and the History of Precision Oncology. Surg. Oncol. Clin. N. Am..

[43] Božović A., Mandušić V., Todorović L., Krajnović M. (2021). Estrogen Receptor Beta: The Promising Biomarker and Potential Target in Metastases. Int. J. Mol. Sci..

[44] Telli M.L., Nagata H., Wapnir I.L., Acharya C., Zablotsky K.E., Fox B.A., Bifulco C.B., Jensen S.M., Ballesteros-Merino C., Le M.H. (2021). Intratumoral plasmid IL-12 expands CD8+ T cells and induces a CXCR3 gene signature in triple-negative breast tumors that sensitizes patients to anti-PD-1 therapy. Clin. Cancer Res..

[45] Yeung C., Hilton J., Clemons M., Mazzarello S., Hutton B., Haggar F., Addison C.L., Kuchuk I., Zhu X., Gelmon K. (2016). Estrogen, progesterone, and HER2/neu receptor discordance between primary and metastatic breast tumors—A review. Cancer Metastasis Rev..

[46] Amir E., Miller N., Geddie W., Freedman O., Kassam F., Simmons C., Oldfield M., Dranitsaris G., Tomlinson G., Laupacis A. (2012). Prospective Study Evaluating the Impact of Tissue Confirmation of Metastatic Disease in Patients with Breast Cancer. J. Clin. Oncol..

[47] Steenbruggen T.G., van Seijen M., Janssen L.M., van Ramshorst M.S., van Werkhoven E., Vrancken Peeters M.-J.T.D.F., Wesseling J., Lips E.H., Sonke G.S. (2019). Prognostic Value of Residual Disease after Neoadjuvant Therapy in HER2-Positive Breast Cancer Evaluated by Residual Cancer Burden, Neoadjuvant Response Index, and Neo-Bioscore. Clin. Cancer Res..

[48] Nowecki Z., Jagiello-Gruszfeld A., Pogoda K., Niwińska A., Olszewski W.P., Winter P., Matkowski R., Wysocki W.M. (2021). Neoadjuvant therapy for breast cancer patients and its impact on surgical treatment and radiotherapy (part 1.). Nowotw. J. Oncol..

[49] Livingston-Rosanoff D., Schumacher J., Vande Walle K., Stankowski-Drengler T., Greenberg C.C., Neuman H., Wilke L.G. (2019). Does Tumor Size Predict Response to Neoadjuvant Chemotherapy in the Modern Era of Biologically Driven Treatment? A Nationwide Study of US Breast Cancer Patients. Clin. Breast Cancer.

[50] Katayama A., Miligy I.M., Shiino S., Toss M.S., Eldib K., Kurozumi S., Quinn C.M., Badr N., Murray C., Provenzano E. (2021). Predictors of pathological complete response to neoadjuvant treatment and changes to post-neoadjuvant HER2 status in HER2-positive invasive breast cancer. Mod. Pathol..

[51] Jagarlamudi K.K., Hansson L.O., Eriksson S. (2015). Breast and prostate cancer patients differ significantly in their serum Thymidine kinase 1 (TK1) specific activities compared with those hematological malignancies and blood donors: Implications of using serum TK1 as a biomarker. BMC Cancer.

[52] He Q., Zhang P., Zou L., Li H., Wang X., Zhou S., Fornander T., Skog S. (2005). Concentration of thymidine kinase 1 in serum (S-TK1) is a more sensitive proliferation marker in human solid tumors than its activity. Oncol. Rep..

[53] Bitter E.E., Townsend M.H., Erickson R., Allen C., O’Neill K.L. (2020). Thymidine kinase 1 through the ages: A comprehensive review. Cell Biosci..

[54] McCartney A., Biagioni C., Schiavon G., Bergqvist M., Mattsson K., Migliaccio I., Benelli M., Romagnoli D., Bonechi M., Boccalini G. (2019). Prognostic role of serum thymidine kinase 1 activity in patients with hormone receptor–positive metastatic breast cancer: Analysis of the randomised phase III Evaluation of Faslodex versus Exemestane Clinical Trial (EFECT). Eur. J. Cancer.

[55] Bonechi M., Galardi F., Biagioni C., De Luca F., Bergqvist M., Neumüller M., Guarducci C., Boccalini G., Gabellini S., Migliaccio I. (2018). Plasma thymidine kinase-1 activity predicts outcome in patients with hormone receptor positive and HER2 negative metastatic breast cancer treated with endocrine therapy. Oncotarget.

[56] Broughton M.N., Westgaard A., Paus E., Øijordsbakken M., Henanger K.J., Naume B., Bjøro T. (2017). Specific antibodies and sensitive immunoassays for the human epidermal growth factor receptors (HER2, HER3, and HER4). Tumor Biol..

[57] Li C., Wang S., Xing Z., Lin A., Liang K., Song J., Hu Q., Yao J., Chen Z., Park P.K. (2017). A ROR1–HER3–lncRNA signalling axis modulates the Hippo–YAP pathway to regulate bone metastasis. Nat. Cell Biol..

[58] Gutsch D., Jenke R., Büch T., Aigner A. (2021). Inhibition of HER Receptors Reveals Distinct Mechanisms of Compensatory Upregulation of Other HER Family Members: Basis for Acquired Resistance and for Combination Therapy. Cells.

[59] Du R., Huang C., Liu K., Li X., Dong Z. (2021). Targeting AURKA in Cancer: Molecular mechanisms and opportunities for Cancer therapy. Mol. Cancer.

[60] Cirak Y., Furuncuoglu Y., Yapicier O., Aksu A., Cubukcu E. (2015). Aurora a overexpression in breast cancer patients induces taxane resistance and results in worse prognosis. J. BUON.

